# Genome-wide analysis of coordinated transcript abundance during seed development in different *Brassica rapa* morphotypes

**DOI:** 10.1186/1471-2164-14-840

**Published:** 2013-12-01

**Authors:** Ram Kumar Basnet, Natalia Moreno-Pachon, Ke Lin, Johan Bucher, Richard G F Visser, Chris Maliepaard, Guusje Bonnema

**Affiliations:** Wageningen UR Plant Breeding, Wageningen University and Research Center, Droevendaalsesteeg 1, 6708 PB Wageningen, the Netherlands; Centre for BioSystems Genomics, PO Box 98, 6700 AB Wageningen, the Netherlands; Wageningen UR Bioinformatics, Wageningen University and Research Center, PO Box 569, 6700 AN Wageningen, the Netherlands

**Keywords:** *Brassica rapa*, Leafy vegetables, Oil-seed, Seed development, Microarray, Co-expression network analysis, Transcript abundance, *cis*-regulatory elements

## Abstract

**Background:**

*Brassica* seeds are important as basic units of plant growth and sources of vegetable oil. Seed development is regulated by many dynamic metabolic processes controlled by complex networks of spatially and temporally expressed genes. We conducted a global microarray gene co-expression analysis by measuring transcript abundance of developing seeds from two diverse *B. rapa* morphotypes: a pak choi (leafy-type) and a yellow sarson (oil-type), and two of their doubled haploid (DH) progenies, (1) to study the timing of metabolic processes in developing seeds, (2) to explore the major transcriptional differences in developing seeds of the two morphotypes, and (3) to identify the optimum stage for a genetical genomics study in *B. rapa* seed.

**Results:**

Seed developmental stages were similar in developing seeds of pak choi and yellow sarson of *B. rapa*; however, the colour of embryo and seed coat differed among these two morphotypes. In this study, most transcriptional changes occurred between 25 and 35 DAP, which shows that the timing of seed developmental processes in *B. rapa* is at later developmental stages than in the related species *B. napus*. Using a Weighted Gene Co-expression Network Analysis (WGCNA), we identified 47 “gene modules”, of which 27 showed a significant association with temporal and/or genotypic variation. An additional hierarchical cluster analysis identified broad spectra of gene expression patterns during seed development. The predominant variation in gene expression was according to developmental stages rather than morphotype differences. Since lipids are the major storage compounds of *Brassica* seeds, we investigated in more detail the regulation of lipid metabolism. Four co-regulated gene clusters were identified with 17 putative *cis*-regulatory elements predicted in their 1000 bp upstream region, either specific or common to different lipid metabolic pathways.

**Conclusions:**

This is the first study of genome-wide profiling of transcript abundance during seed development in *B. rapa*. The identification of key physiological events, major expression patterns, and putative *cis*-regulatory elements provides useful information to construct gene regulatory networks in *B. rapa* developing seeds and provides a starting point for a genetical genomics study of seed quality traits.

**Electronic supplementary material:**

The online version of this article (doi:10.1186/1471-2164-14-840) contains supplementary material, which is available to authorized users.

## Background

*Brassica rapa* (2n = 2x = 20; AA) is an important crop that consists of diverse morphotypes (also called crop types), including oilseed (annual crops yellow sarson and brown sarson, and biannual winter oils), leafy vegetables (Chinese cabbage, pak choi and many non-heading leafy types), turnip (fodder and vegetable turnip) and broccoletto. It contributes the A-genome to the amphidiploid oil crop canola (*B. napus* L; n = 19; AACC). Yellow sarson and brown sarson are grown for oil production in the Indian sub-continent, and in Canada, because of their early maturity and shatter resistance. *Brassica* seed is important for both plant propagation and oil production.

*Brassica* seed is non-endospermic, which means that the endosperm is not retained in mature seeds and only the embryo is enclosed by the seed coat [1]. Seed development goes through basically three overlapping stages: morphogenesis, seed filling and seed desiccation [2, 3]. Embryo development, also known as embryogenesis, starts after the double fertilization process of fusion of two sperm nuclei with the egg cell and the central cell nuclei, respectively, and the zygote goes through a series of cell divisions and differentiation events from a pre-globular and globular embryo stage, a heart stage, a torpedo stage, a bent-cotyledon stage to the mature embryo [3, 4]. Embryogenesis consists of two phases; morphogenesis and seed filling, as the seeds are non-endospermic.

Seed development goes through a complex network of many dynamic developmental, biochemical and metabolic processes such as cell division and differentiation, carbohydrate, protein, cell wall, lipid, amino acid, hormone and secondary metabolite biosynthesis [5]. Several hundreds of genes are reported to be involved in spatial and temporal regulation of these metabolic processes. A systematic overview of metabolic processes and gene expression patterns during seed development has been well documented for the closely related model plant *Arabidopsis thaliana*[5–7]. In *B. napus*, transcript profiling was mainly reported in relation to oil biosynthesis and storage seed reserves [2, 8]. For oil biosynthesis, starch is synthesized at the early seed developmental stage, but after intermediate processes such as malonyl-CoA and fatty acid biosynthesis, converted into triacylglycerol (TAG), lipids and storage proteins during the seed-filling phase at a later stage of seed development in both *A. thaliana* and *B. napus*[5, 9, 10]. A starchless mutant contained up to 40% less lipids in mature *Arabidopsis* seed than the wild-type, while starch was undetectable [11]. Starch turnover, breakdown of cytosolic and plastidic glycolytic pathways, malonyl-CoA and fatty acid (FA) synthesis, TAG assembly and oil body formation takes place during TAG synthesis in seed [9]. The plant hormones gibberellin, auxin, ethylene and abscisic acid (ABA) play key regulatory roles in seed development and growth [12, 13] and changes in hormonal levels affect the seed size and seed number in *B. napus*, especially during the 10–20 days after pollination (DAP) period [14]. Transcription factors, for example, ABI3 (Abscisic acid insensitive-3), ABI4, ABI5, LEC1 (leafy cotyledon1), LEC2 and FUS3 (FUSCA3) are important regulators of the complex gene network during the process of seed development, maturation and germination [15, 16].

Understanding the regulatory mechanisms of seed development is essential to identify the molecular basis of seed development. Transcript profiling of developing seeds has been a widely used strategy to identify functional genes and their regulatory elements for seed development that can be used as tools in breeding programs for seed quality traits. Transcriptomics provides a powerful tool and is widely used to examine the temporal and spatial changes in transcript abundance during seed development in *Arabidopsis*[4, 7, 10, 17], *B. napus*[2, 18, 19], wheat [20, 21], maize [22, 23], barley [24], rice [13, 25], soybean [26], *Jatropha*[9] and many other crops. So far, we are not aware of any studies connecting global gene expression profiles to seed developmental stages in the diploid *Brassica* species *B. rapa*. The release of the whole-genome sequence of *B. rapa* morphotype Chinese cabbage var. Chiifu [27] facilitates genomic studies, such as gene expression analysis and genetical genomics studies [28]. The knowledge on changes in gene expression associated with specific stages of seed development is crucial to unravel the molecular and biochemical events that influence optimal seed metabolite composition [29]. Timing of major transition stages differs between metabolic pathways (carbohydrates, fatty acids, storage proteins) and also between species. The higher number of differentially expressed sequence tags (ESTs) at 15 DAP than at 25 DAP in *B. napus* suggest that most developmental changes take place at 10–20 DAP [18]. Major changes in gene expression profiles of genes involved in protein translation, starch metabolism and hormonal regulation were reported between 17–21 DAP in *B. napus*, whereas fatty acid synthesis related genes were highly expressed at 21 DAP as compared to earlier and later time points [10]. In developing *B. napus* spring cultivar seeds, 20 DAP was the most active stage to measure variation in transcript abundance of genes related to the biosynthesis of starch, lipids, carotenoids, isoprenoids, proteins and storage reserves [2].

Recently, genetical genomics has become a powerful tool to find candidate genes for complex traits [28, 30], such as seed quality and seedling vigour traits [31]. In this approach variation in transcript abundance is considered as quantitative traits in quantitative trait loci (QTL) analyses per gene, resulting in identification of genomic regions regulating gene expression (called expression quantitative trait loci: eQTL). It is important to find an optimum stage during seed development for eQTL mapping studies, where large numbers of genes show differences in transcript abundance between genotypes in a segregating population. To obtain a comprehensive insight into transcriptional changes during seed development in *B. rapa*, we carried out morphological characterization and global transcriptome analysis in a time range of developing seeds of a black/brown-seeded pak choi vegetable-type (PC175), a yellow-seeded oil-type yellow sarson (YS143) and both a yellow and a black/brown-seeded doubled haploid (DH) progeny line from their cross. In this study, we first describe embryo and seed morphological changes in time. Second, the differential expression profiles of genes from different metabolic pathways and transcription factors in developing seeds of the four genotypes are presented. Third, a window around the optimum seed development stage was defined based on genotypic and developmental transcriptomic profiles for more extended gene expression studies. Fourth, we investigated the regulation of lipid metabolism in more detail. Using a comparative analysis of gene expression networks among these four different genotypes, we explore the differential gene expression profiles and conserved regulatory mechanisms for seed development across these morphotypes of the diploid crop species *B. rapa*.

## Results

### Morphology of developing seeds and embryos

**Figure 1:**
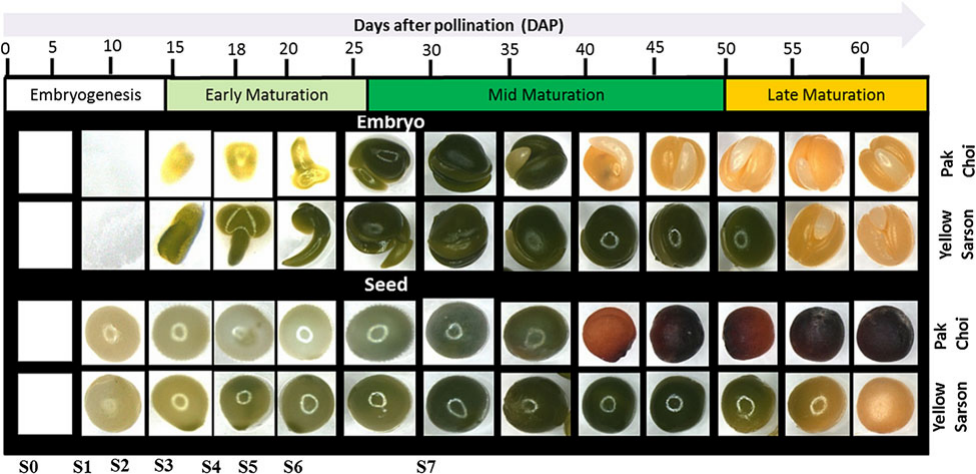
**Morphological characterization of embryos and developing seeds of yellow-seeded oil-type genotype yellow sarson (YS143), and black/brown-seeded vegetable-type genotype pak choi (PC175) of** ***Brassica rapa*** **.** Developing seeds represent different developmental stages after fertilization to seed maturity. Seed developmental stages are: S0: fertilization; S1: pre-globular; S2: globular; S3: heart; S4: torpedo; S5: linear cotyledon; S6: bent-cotyledon; S7: embryo fully fills seed.

Morphological changes in developing embryos and seeds were monitored from 10 DAP until 60 DAP. The images show that seed and embryo structure were visible at 10 and 15 DAP, respectively (Figure 1). The colour of the embryo in YS143 was green already at 15 DAP (torpedo stage) and changed from green to yellow at 55 DAP, while in PC175 the embryo turned green only around 25 DAP (bent-cotyledon) and changed from green to yellow at 40 DAP (embryo fully fills seed) (Figure 1). In the case of the seed coat, the colour gradually turned from pale yellow to greenish or green until 40 DAP, then turned to brown or black in pak choi; however, for YS143 the seed coat colour changed from green to yellow from 55 DAP (Figure 1). Different embryo developmental stages could be defined in time, such as: pre-globular, globular, heart shape (<15 DAP), torpedo (15–18 DAP), bent-cotyledon (20–30 DAP), embryo filling seed completely (30–40 DAP) (Figure 1).

### Real-time gene expression profiling in developing seeds

The transcript abundance of 10 selected genes from a few key metabolic processes and transcription factors (Additional file 1: Figure S1) was measured from 10 to 60 DAP to obtain an overview of gene expression patterns during seed development. Three patterns were observed, with peak levels at 10–25 DAP, 25–40 DAP and 35–60 DAP which are defined as early-, mid- and later- stage, respectively (Additional file 1: Figure S1). These patterns were not very different among the four genotypes tested. Out of the ten genes, transcription factors LEC1 and Glabra2 and the starch gene GBSSI were expressed higher during earlier stages, lipid metabolism genes DGAT2 and FAE1, and storage protein 12S-CRA1 were expressed higher at mid stages, while the lipid metabolism gene DGAT1 (also called TAG1), carbohydrate metabolism gene SUS3, and the storage protein LEA and CHD3-chromatine-remodeling factor PICKLE were expressed highest at late stages. For the whole genome microarray gene expression profiling, six time points were selected: (i) 18 DAP (torpedo), (ii) 20 DAP (bent-cotyledon), (iii) 25 DAP (transition bent-embryo fully fills seed), and the developmental stages where the embryo fully fills the seed, being (iv) 30 DAP (v) 35 DAP and (vi) 40 DAP. These time points captured transcriptional changes at early, mid and late stages of seed development.

### Microarray hybridization and probe annotation

In a dedicated *B. rapa* Agilent array, 61,546 probes (99.7% of total 61,654 probes) represent 42,162 *Brassica rapa* gene ID (called Bra ID). Out of 42,162 Bra IDs, 30,363 Bra IDs (72%) were assigned to 34 MapMan functional annotation categories. The remaining 11,799 (28% ) Bra IDs were not assigned to any functional category (Additional file 2: Table S1).

Pearson correlation coefficients were calculated to quantify how similar transcript abundance was between time-points and also between four replicates in each genotype (YS143, PC175, DH42 and DH78). All the replicates of each genotype from each time point had high correlations (r > 0.95) in all four genotypes (Additional file 3: Figure S2A-B). The correlation coefficients between time points decrease as the time points increase. Pearson correlation coefficients of transcript abundance between time-points were high (r > 0.9) from 18 to 25 DAP in PC175, and from 18 to 30 DAP in YS143, DH42 and DH78, but after those time points a transition from high (r > 0.95) to lower (r < 0.85) correlation coefficients occurs between early and later time points.

### Correlation of transcript abundance of genes from real-time PCR and microarray analysis

Since transcript abundance was measured using two different techniques: qRT-PCR and microarray that might lead to a non-linear relationship, Spearman’s rank correlation coefficients, which are free from parametric assumptions, were used to compare the outcome of these two techniques. The transcript abundance from qRT-PCR and microarray of 10 selected genes were significantly and positively correlated except for transcription factors LEC1 and CHD3-chromatine-remodeling factor PICKLE. The rank correlation coefficients ranged from 0.43 for DGAT2 to 0.94 for LEA protein (Additional file 4: Table S2).

### Genome-wide variation in transcript abundance during seed development

**Figure 2:**
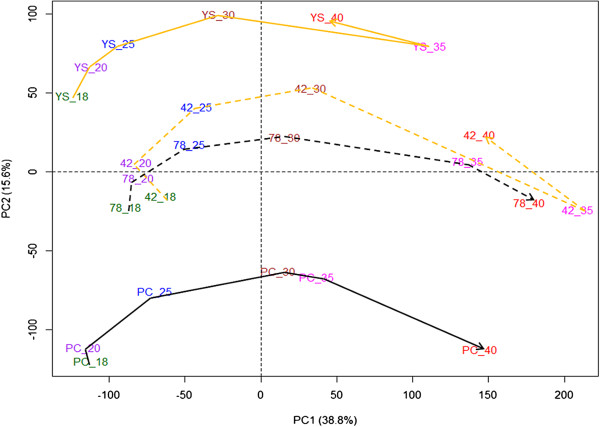
**Principal components analysis (PCA) of two parental genotypes (YS143 and PC175) and two DH lines (DH42 and DH78) based on transcriptional profiles during seed development (18–40 DAP).** Sample names are a combination of genotypes (YS = yellow sarson, PC = pak choi, 42 = DH line 42 and 78 = DH line 78) and time points in days after pollination (DAP). The yellow lines represent yellow-seeded genotypes YS143 and DH42, and black lines represent black/brown-seeded genotype PC175 and DH78. Parental genotypes are indicated with solid lines, and DH lines with dashed lines. Sample labels were coloured according to time points: 18 DAP - green, 20 DAP - purple, 25 DAP - blue, 30 DAP - brown, 35 DAP - pink and 40 DAP - red.

Principal components analysis (PCA) on transcript abundance of the 61,654 probes showed a sequential distribution of the six time points according to seed developmental stages along the first principal component (PC1) and separation of the four *B. rapa* genotypes along PC2. PC1 explained 38.8% of total variation, and is associated mostly with variation in transcript abundance over the developmental stages, where 18 DAP and 20 DAP form a tight group, with 25 DAP more loosely grouped with these earlier stages. Similarly, 35 DAP and 40 DAP were grouped together (but distinct from the earlier time points) except in PC175. Major changes in transcript abundance were observed between 25 and 35 DAP (Figure 2), which coincides with the period of transition from bent-cotyledon to the stage when the embryo fully fills the seed. PC2 explained 15.6% of the total variation, and reflects mostly genotypic differences. Interestingly, the two DH lines were grouped in between the two parental genotypes (Figure 2).

We investigated the loading values of probes on PC1, where probes with very low negative loadings were associated with the early stage of seed development (18–25 DAP) while the probes with very high positive loading were in response to later stages (35–40 DAP) (Figure 2). Among 34 MapMan functional categories, probes with high positive or low negative loadings mainly belong to metabolic pathways such as photosynthesis, cell wall metabolism, lipid metabolism, amino acid metabolism, protein metabolism, signalling, RNA (RNA processing, RNA binding and transcription factors), stress, transport, developmental processes, hormone metabolism, phosphate metabolism and secondary metabolism (Additional file 5: Figure S3).

### Apparent changes in numbers of selected probes in contrasts between developmental stages or genotypes lead to selection of metabolic pathways

After excluding probes with rather constant transcript levels (< 2-fold change) across seed development and between genotypes, 11,244 probes (18.2% of total 61,554 probes) were retained for further analysis (Additional file 6: Table S3). Based on either a high number of selected probes per pathway or apparent changes in the number of selected probes from contrasts between consecutive time points or between genotypes at each time point, the top thirteen metabolic pathways were emphasized in this study. These top thirteen metabolic pathways correspond to metabolic pathways highlighted based on higher PC1 and PC2 loadings in PCA analysis. Those top thirteen metabolic pathways are represented by 9606 probes (i.e. 5520 Bra ID) and used for network analysis to separate the gene clusters according to temporal (4178 probes) and/or genotypic variation (3169 probes) during seed development (Additional file 7: Table S4).

### Signed weighted gene co-expression network analysis (WGCNA) identifies gene modules associated with temporal and or genotype effects

Signed WGCNA grouped the selected probes (> 2 fold-change) into 47 co-expression gene modules, each one containing probes with a similar transcript abundance across genotypes and seed developmental stages. In an analysis of variance (ANOVA) test, 17 gene modules (3169 probes) showed a genotype effect, 4 modules (4179 probes) a time effect, and 6 modules (555 probes) a genotype as well as a time effect at 0.001 significance level and the remaining 20 gene modules did not show any effect (Additional file 8: Table S5; Additional file 9: Figure S4A-C). Since some of the gene modules showed similar expression patterns with subtle differences, gene modules were combined according to the time or genotype or time and genotype effects, and subjected to hierarchical clustering to have a broader overview of the patterns of transcript abundance.

### Temporal variation across seed development stages

**Figure 3:**
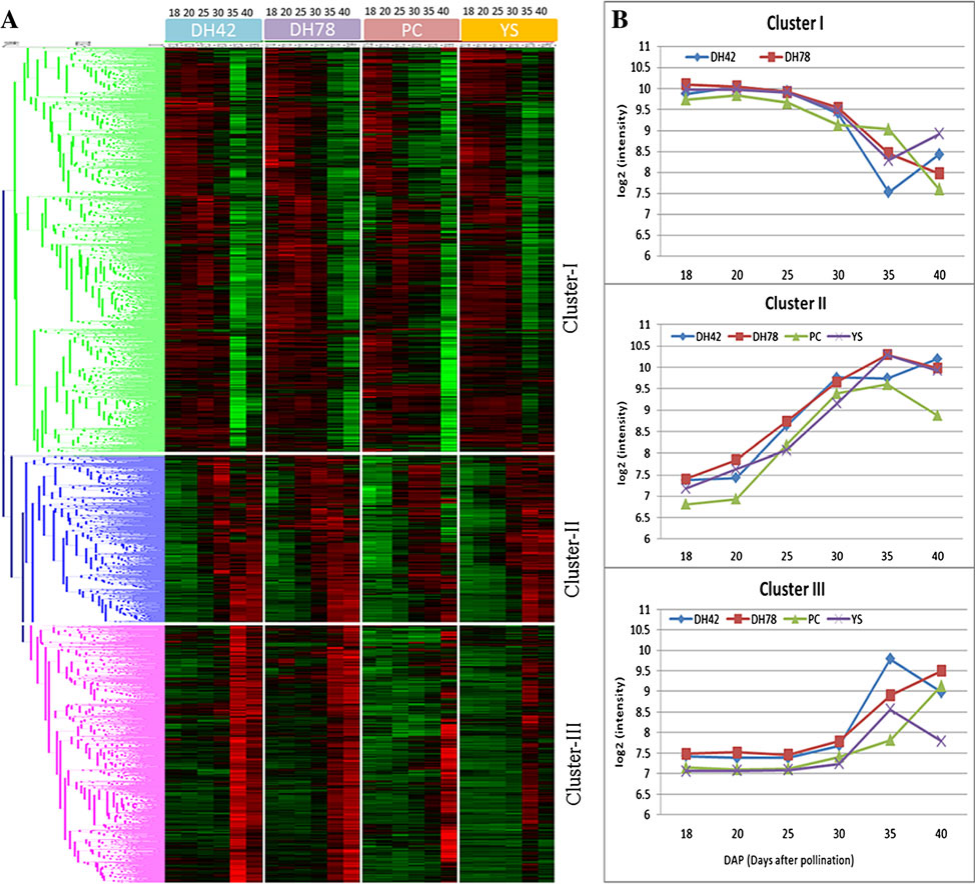
**Temporal patterns of transcript abundance during seed development stages (18-40 DAP). A**. Hierarchical cluster analysis using Euclidean distance and average linkage of all probes belonging to four WGCNA gene modules having a significant effect of developmental stages. Vertical white bars separate genotype and horizontal bars separate gene clusters. Red colour indicates a higher level of transcript abundance, green colour lower abundance and black an intermediate level. Colours of dendrogram branches indicate different gene clusters. **B**. Line graph that shows the expression level (log_2_ scale) of probes that belong to three clusters. The x-axis represents seed development time points (18, 20, 25, 30, 35 and 40 DAP: Days after pollination).

Using hierarchical clustering, 4179 probes from the four gene modules (associated with differential expression in time) were classified into three clusters (Figure 3A-B). Cluster I (2043 probes corresponding to 1525 genes) represents genes with higher transcript levels at earlier stages (18–25 DAP) from linear cotyledon to bent-cotyledon. Both cluster II (837 probes or 655 genes) and cluster III (1298 probes or 977 genes) show increased transcript abundance in time, from 18 DAP for cluster II and from later stages after the embryo fills the seed at 30 DAP for cluster III (Figure 3A-B).

**Figure 4:**
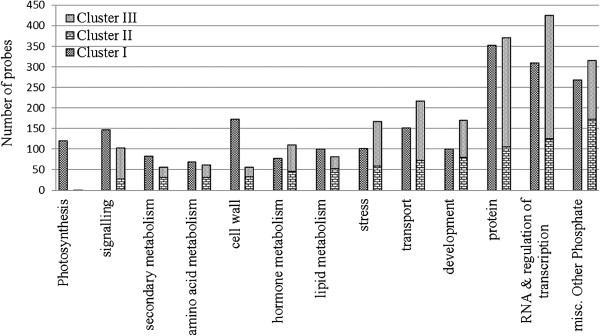
**Comparison of numbers of probes belonging to MapMan functional categories in three clusters (Cluster I, cluster II and cluster III) showing temporal variation of transcript abundance.** Fisher’s exact test was carried out for over-representation against total numbers of probes annotated in each functional category. The significance level was determined at 0.01 p-value after FDR correction with the method of Benjamini-Hochberg (1995). “ns” indicates “non-significant” in Fisher’s exact test.

Genes associated with photosynthesis (Calvin cycle and photosystem-I and –II), Fatty acid (FA) synthesis, FA elongation and lipid degradation are over-represented only in cluster I, so, these genes are active early in seed development and down-regulated later (Figure 4; Additional file 10: Figure S5). Also genes from tocopherol biosynthesis, mevalonate and carotenoids in secondary metabolism, as well as from biosynthesis of serine, glycine, cysteine, glutamate, aspartate and alanine amino acids were only over-represented in cluster I. Transcription factors (TFs) were mostly under-represented in this cluster I. For example, AP2/EREBP, bHLH, C2H2, myb, and WRKY TFs were under-represented in cluster I, and bZIP was overrepresented in cluster II. Genes involved in cell wall metabolism including precursor synthesis, cellulose synthesis, cell wall proteins and cell wall degradation, and genes in triacylglycerol synthesis (TAG) and FA desaturation were mainly over-represented in cluster II, which means that they continuously increase in abundance from 18 DAP till 35 DAP. Also, storage protein genes and genes related to the biosynthesis of auxin, brassinosteroid and gibberellin and branched-chain and aromatic amino acids (Additional file 10: Figure S5) were over-represented only in this cluster II. Similarly, metabolite transporter genes and major intrinsic protein genes from transport metabolism were mainly over-represented in both clusters I and II, but receptor kinases and G-proteins genes from signaling pathway, and genes involved in protein synthesis, protein posttranslational modification, protein degradation, RNA processing and RNA binding were under-represented in cluster I and or II. Genes related to cytochrome P450 and seed storage (lipid transfer protein, LTP) of phosphate metabolism, late embryogenesis abundant (LEA) proteins, and ethylene and abscisic acid from hormonal metabolism were over-represented in cluster II and or III, so their abundance increased during seed development. Biotic stress tolerance genes related to PR-proteins were underrepresented in cluster II and III but genes related to heat shock proteins for abiotic stress tolerance were overrepresented in cluster III. Interestingly, cluster II and III had high transcript abundance during late stages of seed development with different patterns.

### Putative *cis*-regulatory elements underlying co-expressed genes of lipid metabolism

We looked in more detail to changes in transcript abundance related to lipid metabolism because oil is the major storage compound of *Brassica* seeds. *B. rapa* and *B. napus* are widely grown for oil production, while *B. rapa* is also grown as vegetable crop. Therefore, it is interesting to know the variation in transcript abundance of genes related to oil biosynthesis during seed development in oil-type and non-oil type morphotypes. For this study, two genotypes: a yellow-seeded oil-type genotype YS143 and a black/brown-seeded vegetable-type genotype PC175 were chosen. In addition, two DH progeny lines, a yellow-seeded and a black/brown-seeded line, resembling the two parental lines were also used to develop ideas on segregation of transcript abundance of oil biosynthesis genes. In Additional file 11: Figure S6, the pathway for oil biosynthesis is depicted, with acetyl-CoA as the main precursor for the synthesis of fatty acids (FA), triacylglycerol (TAG) and phospholipids. Transcript abundance was visualized separately for genes involved in FA synthesis, FA elongation, lipid degradation, FA desaturation, biosynthesis of TAG and phospholipids, and oleosin (oil bodies).

In the process of FA synthesis and elongation, transcript abundance of genes revealed patterns with either a clear temporal effect or with a clear genotype effect (Additional file 12: Figure S7A:I-II). Transcript abundance of 63% of FA synthesis and FA elongation related probes was high at early stages (18–30 DAP), followed by a gradual decrease, while other probes (37%) show clear genotype differences with higher transcript abundance in the two progeny lines (DH42 and DH78) as compared to the parental genotypes. FA desaturation genes such as ADS1, FAD6 and FAD7 were up-regulated before 30 DAP, but FAD3 and ADS2 genes including FAD6 and FAD7 paralogs were up-regulated after 25 DAP (Additional file 12: Figure S7B). Triacylglycerides are the main constituents of vegetable oil and expressed at late stages of seed development. Genes involved in triacylglycerol biosynthesis, such as DGAT-1 and −2, GRP (glycine rich protein) and oleosin (storage proteins) were mainly up-regulated after 25 DAP (Additional file 12: Figure S7D).

For lipid degradation, four different patterns of transcript abundance were observed. A set of probes (18.5%) had high transcript abundance at later stages of seed development (after 25 DAP) (Additional file 12: Figure S7C: I-IV), while a larger number of probes (40.7%) showed higher transcript abundance at earlier stages before 30 DAP (Additional file 12: Figure S7C: II). Additional file 12: Figure S7C: III consists of a set of probes (13%) with high transcript abundance only at 35 and 40 DAP. Probes (27.8%) from Additional file 12: Figure S7C: IV showed genotype differences in transcript abundance with lower levels in parental genotypes PC175 and YS143, than in the DH lines.

A set of the genes functionally related and/or co-expressed often share conserved regulatory motifs, which might be responsible for coordinated expression of the set of genes. In this study, genes related to lipid metabolism with different co-expression patterns (different clusters) were searched to computationally predict *cis*-acting regulatory elements for potential roles in regulating lipid metabolism during seed development in *B. rapa* species. For all the selected 194 *B. rapa* genes (> absolute 2-fold change), the 1000 bp upstream sequence from the gene start were retrieved.

**Figure 5:**
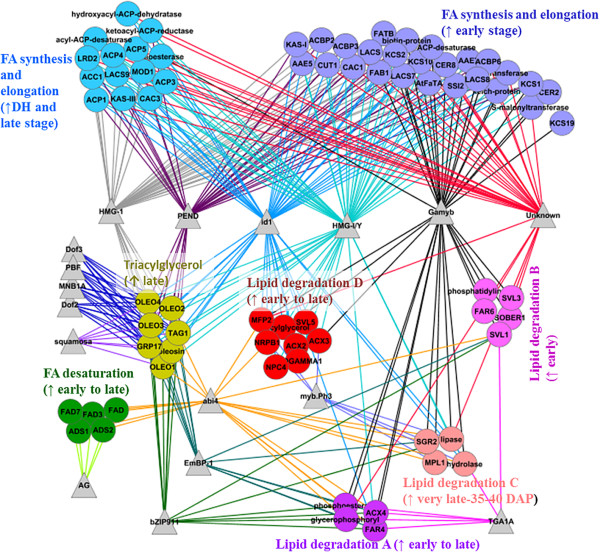
**Graph showing motifs (TFBS: transcription factor binding sites) identified in sets of co-expressed genes from different metabolic processes of lipid metabolism.** The elliptic shaped node represents genes, the triangular node represents conserved motifs, the edge between motif and gene represents the presence of a motif in a particular gene. The colour of nodes indicates co-expressed genes from different metabolic processes of lipid metabolism while the same colour of the edges indicates genes have same motif. An arrow-up symbol indicates high transcript abundance of a gene.

In total, 17 regulatory motifs were predicted for FA synthesis and elongation (92 genes), lipid degradation (74 genes), lipid desaturation (12 genes) and triacylglycerol (16 genes) processes considering gene clusters with comparable patterns in transcript abundance (Table 1; Figure 5). Co-expressed gene clusters from the FA synthesis and elongation, and lipid degradation, and/or other lipid metabolic processes shared most of the motifs (Figure 5). Each TF (transcription factor) can have more than one putative binding site in each gene. The DOF motif family, including DOF2, DOF3, PBF and MNB1A, and MADS motif-squamosa were specific to the TAG biosynthesis process but another MADS motif – AG was specific to FA desaturation. TGA1A (leucine zipper family) and myb.Ph3 (myb family) were shared among different co-expression groups of lipid degradation genes. The ABI4 transcription factor binding site was present in genes involved in TAG biosynthesis, FA desaturation and different co-expression groups of lipid degradation, which had high transcript abundance at late stages (after 25 DAP) (Figure 5; Additional file 12: Figure S7A-D). We did not find any motif that is specific to the FA synthesis and elongation process. However, six motifs; HMG-1, HMG-I/Y, PEND, id1, Gamyb and four unknown motifs were shared between two co-expression groups of FA synthesis and elongation genes along with genes from other processes. Conserved motifs that were not significantly overrepresented in plant-specific TFs databases are here indicated as “unknown”. Motifs such as, HMG-1 and PEND were specific to only genes involved in TAG biosynthesis, and FA synthesis and elongation process. Similarly, Gamyb (myb-family) and unknown motifs were specific to only lipid degradation and FA synthesis and elongation process. Motifs- bZIP911 and EmBP-1 from the leucine zipper family were shared among genes from TAG biosynthesis and lipid degradation (Table 1; Figure 5).

**Table 1:** **List of overrepresented motifs identified in promoter regions (1000 bp upstream) of genes involved in FA synthesis and elongation, FA desaturation, FA degradation and triacylglycerol (TAG) synthesis**

Sequence logo	Matrix ID	TFBS name	TFBS family
	MA0123.1	abi4	AP2 MBD-like
	MA0021.1	Dof2, Dof3, MNB1A, PBF	Dof
	MA0097.1	bZIP911	Leucine Zipper
	MA0129.1	TGA1A	Leucine Zipper
	MA0128.1	EmBP-1	Leucine Zipper
	MA0127.1	PEND	Leucine Zipper
	MA0005.1	AG	MADS
	MA0082.1	Squamosa	MADS
	Unknown	Unknown	Unknown
	Unknown	Unknown	Unknown
	Unknown	Unknown	Unknown
	Unknown	Unknown	Unknown
	MA0054.1	myb.Ph3	MYB
	MA0034.1	Gamyb	MYB
	MA0045.1	HMG-I/Y	High mobility group
	MA0044.1	HMG-1	High mobility group
	MA0120.1	id1	Zinc finger

### Genotypic variation in overall metabolism

**Figure 6:**
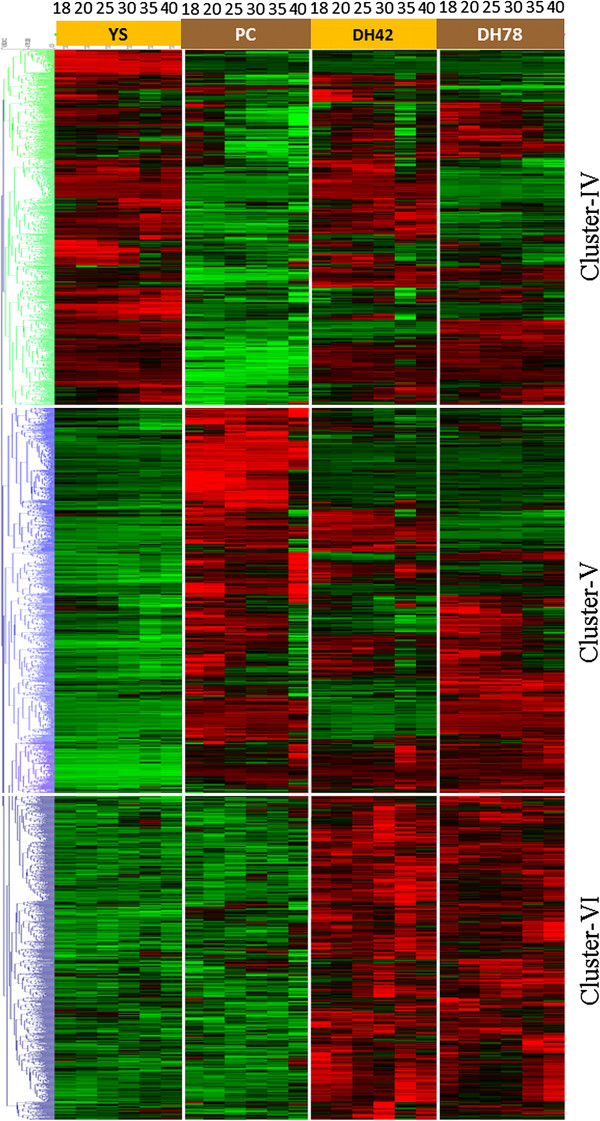
**Hierarchical cluster analysis (Euclidean distance; average linkage) on all probes from four WGCNA gene modules with significant genotypic effects.** Vertical white lines separate genotypes and horizontal white lines separate gene clusters. The bright red to bright green colour represent high to low abundance levels, black for an intermediate level of abundance.

In total 17 modules (3169 probes) were divided into three clusters (cluster IV to VI) in hierarchical cluster based on clear contrasts in patterns of transcript abundance only between the two parental genotypes. Probes from cluster IV (1054 probes, 851 genes) were up-regulated in YS143 and down-regulated in PC175, while probes in cluster V (1149 probes, 951 genes) had higher transcript abundance in PC175 and lower in YS143 (Figure 6). These two clusters differentiate the transcript abundance between the two parental genotypes. However, the two DH lines had a mixture of levels of transcript abundance. In contrast, genes belonging to cluster VI (966 probes, 878 genes) had low transcript abundance in both parents but high in the two progeny DH lines. Genes mainly involved in the synthesis and degradation of amino acid, cell wall, hormones, lipids, isoprenoids and ion transport, and also different transcription factors were significantly over- or under- represented in those three clusters (Additional file 13: Figure S8).

### Genotypic as well as temporal variation in overall metabolism

**Figure 7:**
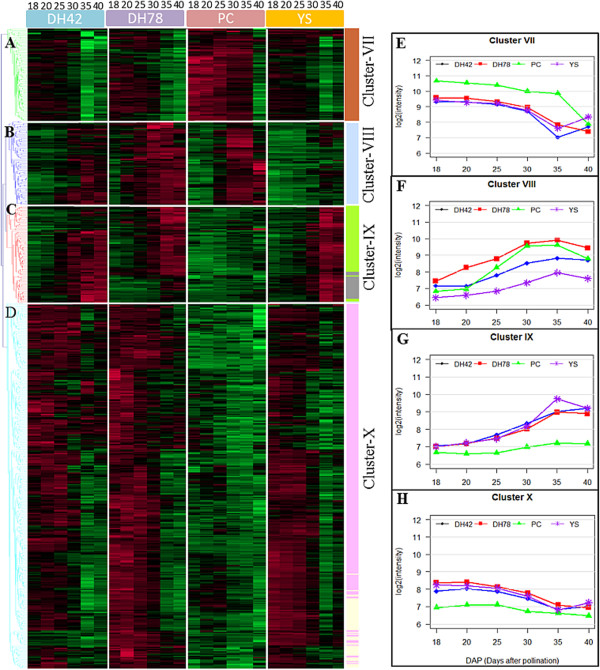
**Characteristics of transcriptional patterns in seed development stages (18 – 40 DAP) showing genotypic and temporal variation. A-D** Hierarchical clustering (Euclidean distance, average linkage) on 555 probes from six WGCNA gene modules with both a genotype and time effect. Vertical white bars separate genotype and horizontal white bars separate cluster of genes. Colours of dendrogram branches indicate different clusters of genes while the colour bar on the right side indicates WGCNA gene modules. Red indicates high, green low, black an intermediate level of transcript abundance. **E-H** mean abundance of transcripts on four genotypes (YS143, PC175, DH42 and DH78) representing gene clusters **A-D** respectively.

Six WGCNA modules (555 probes) showed both significant genotypic and temporal variation in ANOVA (Additional file 8: Table S5) and four clusters of probes with different patterns of transcript abundance were observed in a hierarchical cluster analysis (cluster VII to X; Figure 7A-H). Genes in cluster VII (80 probes, 77 genes) reached their maximum at 18 DAP, gradually decreasing until 35 DAP (YS143 and DH42) or 40 DAP (PC175 and DH78). Transcript abundance in PC175 was always higher than in other genotypes except at 40 DAP (Figure 7A and 7E). Transcript abundance of genes from cluster VIII (73 probes, 64 genes gradually increased until 35 DAP and then started to decrease in all genotypes. Transcript abundance in DH78 was highest while it was lowest in YS143 across all the time points. Transcript abundance in PC175 was lower than in DH42 during 18–20 DAP but increased to the level of DH78 during 25 DAP to 35 DAP (Figure 7B and 7F). Genes in cluster IX (85 probes, 76 genes) had similar transcript abundance compared to cluster VIII with a gradual increase across the developmental stages till 35 DAP which then remained constant. However, genes from cluster IX had a lower transcript abundance in PC175 across time (Figure 7C and 7G). A larger number of probes (317 probes, 267 genes) were grouped in cluster X, which showed a maximum at the earlier stages 18–20 DAP, and then a gradual decrease until 35 DAP from which time point it remained at a constant level (Figure 7D and 7H). This transcript abundance was similar to that of cluster VII except for PC175. Among the four genotypes across all the time points, cluster X genes had the lowest transcript abundance. These clusters indicate the occurrence of major changes in the transcription profiles between the bent-cotyledon to the fully-developed embryo stages of seed development (25–35 DAP).

## Discussion

The understanding of morphological and transcriptional changes during seed development has fundamental applications in *Brassica* breeding, both for high quality vegetable oil content and for crop establishment. In this study, we focused on analysis of morphological characteristics and global transcriptome analysis in developing seeds of four genotypes including two diverse *B. rapa* morphotypes: a leafy-type pak choi and an annual yellow-seeded oil-type yellow sarson. We also predict putative regulatory elements for lipid metabolism to understand this complex regulatory network during seed development.

### Seed morphology varies at the later stages of seed development

Seed developmental stages, which are defined based on the shape of the embryo, were similar in both YS143 and PC175, irrespective of apparent differences in phenological characteristics, such as flowering time or seed colour in the two distant morphotypes pak choi and yellow sarson (Figure 1). However, the colour of embryo differed among these two genotypes at early stages (in period 15–25 DAP, PC175 embryos are yellowish, while YS143 embryos are green); and at later stages (at 40 DAP PC175 embryo’s turn from green to yellow, while YS143 embryo’s turn yellow only at 55 DAP). Also, seed coat colour changes differed among these two morphotypes, as the seed coat of PC175 turns from green to brownish at 40 DAP, while the YS143 seed coat turns yellowish at 50 DAP (Figure 1). Also in the two DH lines, the black/brown-seeded line DH78 lost the green colour earlier than the yellow-seeded DH42. Yellow seed colour is a desired quality trait in breeding *Brassica* oilseed species, because of its association with higher oil content and more easily digestible seed meal as compared to dark coloured seeds. The accumulation of proanthocyanidins (PAs) in the seed coat of immature black/brown seeds (20 DAP) but not in yellow seed [32] might be an explanation for the earlier change in seed colour. In this study, we observed that the embryo completely filled the seed at the bent-cotyledon stage (30 DAP); also Li et al., [3] described that this stage was not yet reached at 25 DAP, but fully reached at 35 DAP in *B. campestris* (Synonymous: *B. rapa*). *Brassica* seed is non-endospermic, so, the endosperm is not retained in mature seeds, but only the embryo is enclosed by the seed coat [1]. Evaluation of transcript abundance using real-time PCR was effective to define six time-points when abundance levels of a set of genes representative for the seed filling process varied with respect to their morphology: 18 DAP (torpedo), 20 DAP (bent- cotyledon), 25 DAP (transition bent-embryo fully fills seed) and 30 DAP, 35 DAP and 40 DAP (embryo fully fills the seed).

### Seed developmental stages are the predominant cause for variation in transcript abundance

Genome-wide transcriptome analysis was used to explore global gene expression at six time points as representative stages for seed development in four genotypes of *B. rapa*. Despite the fact that *B. rapa* is an important vegetable and/or oil crop, this is the first study in which transcript abundance was profiled genome-wide during seed development in this species. The availability of whole genome sequence of *B. rapa*[27] facilitated the design of a 60-mer oligonucleotide microarray platform (62,654 probes targeting 42,162 *Brassica* genes) based on predicted gene models from the genome sequence.

We used four approaches to define sets of genes with different transcript abundance during seed development in time (developmental stages) or between genotypes or both. First PCA was used to obtain an overview of variation in seed developmental stages and also between different genotypes using all the transcripts present in the microarray (Figure 2). The first principal component (PC1: 38.8% explained variance) captured mostly temporal variation in transcript abundance, supporting the earlier findings that seed developmental stages are major sources of transcriptional and metabolic variation in *Arabidopsis*[7, 33]. A comparative study of the transcript and metabolite profiles in both wild-type and transgenic genotypes of *Arabidopsis* also showed more variation across seed developmental stages than changes due to genotypic differences [34]. The genotypic variation was captured in PC2 (15.6% explained variance), which suggests that metabolic processes inside developing seed are largely conserved, even between yellow-seeded oil and black/brown-seeded genotypes. Secondly, we selected a subset of genes with variation in transcript abundance patterns between developmental stages as well as between genotypes based on PCA loadings with a minimum two fold change criterion for further analysis. These subsets of genes represent the most active metabolic processes occurring in *B. rapa* developing seeds, such as photosynthesis, hormonal regulation, stress tolerance, cell wall, lipid, phosphate, amino acid, protein, signal transduction, transport, secondary metabolites, developmental process, and RNA processing and regulation of transcription (Additional file 7: Table S4). Those selected metabolic processes were also reported as major metabolic processes during seed development in close relatives *A. thaliana*[17] and *B. napus*[2], but also in maize [35]. Thirdly, a WGCNA approach was used to discover possible modules consisting of groups of genes with similar transcript abundance, either across time or between genotypes of both, and 27 modules out of a total of 47 modules showed significant variation in transcript abundance across time points or genotypes or their combinations (Additional file 8: Table S5; Additional file 9: Figure S4A-C). Since WGCNA uses Pearson correlation coefficients to identify co-expressed modules, it could not group genes that have similar patterns of transcript abundance but different levels into separate modules. So, in addition a separate hierarchical clustering using Euclidean distance was done in all gene modules according to the type of effects. The combined analysis using both Pearson correlation coefficients with WGCNA and hierarchical clustering with Euclidean distance resulted in clusters that are both similar in transcript abundance and level among genotypes across time points. Finally, we focused on transcriptional profiling related to lipid metabolism, in order to correlate co-expression patterns within pathways and to predict putative regulatory elements of lipid metabolism.

### Global variation in transcript abundance: 25–35 DAP is a key period for major changes in *B. rapa* developing seed

In PCA, the early time points, before the embryo fills the seed (25 DAP), cluster tightly in PC1 but the later time points (35–40 DAP) cluster loosely, suggesting that physiological processes differentiate more at later stages. Higher correlations (r > 0.9) between the early time-points within genotypes and decreasing correlations between later stages also supports that there is more variation in transcript abundance at later stages (after 25 DAP) than at earlier stages (Additional file 3: Figure S2A-B). Variation in metabolite content, seed maturity, desiccation and dormancy induction occurred during the maturation phase [1], which corresponds to 25 DAP in this study. Interestingly, sequential changes in transcript abundance follow developmental changes in the black/brown-seeded genotypes (PC175 and DH78) but an extreme shift from 30 to 35 DAP and reversed at 40 DAP occurred in yellow-seeded genotypes (YS143 and DH42). This signifies the different transcriptome signatures of seed development in different genotypes, especially at the later stage. These findings are in agreement with a different timing of seed and embryo colour changes from 40 DAP onwards (Figure 1). The spatial position of the two DH lines between the two distant parental genotypes in the PC2 dimension points to variation in transcript abundance that can be used for genetic studies.

The largest changes in transcript abundance during seed development were observed during 25–35 DAP (bent-cotyledon to stage when embryo fully fills the seed), suggesting that this is the most optimal stage for genetical genomics studies for mapping eQTL in *B. rapa* developing seeds. In contrast, for *B. napus*, the major transcriptional transition was reported to be much earlier during heart-shaped to torpedo embryo stages i.e. 17–21 DAP, and for FA synthesis-related genes at 21 DAP in a spring and winter type *B. napus* L. cv HuYou15 [29].

### Temporal changes in transcript abundance conserved across different morphotypes

The WGCNA method is a powerful and widely used tool to identify co-expressed gene clusters and to construct scale-free networks using topological properties of network construction [36]. Among 47 gene modules identified, four (4179 probes) show temporal variation in transcript abundance across seed development (Additional file 8: Table S5, Additional file 9: Figure S4B), and these were reduced to three clusters after hierarchical clustering using Euclidean distance (Figure 3). This result, like PCA, confirms that variation in transcript abundance during seed development is predominantly conserved across genotypes in *B. rapa*. Similar observations were made for FA biosynthesis genes, which were conserved between *B. napus* and *A. thaliana*[10]. The annotations of many genes belonging to these three clusters fitted what is known about different processes occurring during seed development. Among the three clusters, cluster I (48% genes) had high transcript abundance before 25 DAP with a gradual decrease till 35 DAP, with genes involved in photosynthesis, secondary metabolic pathways, and biosynthesis of tocopherols, mevalonate and carotenoids, and amino acids were over-represented. Amino acids are known as essential precursors for biosynthesis of secondary metabolites, proteins and other metabolic biosynthetic processes. Tocopherols are fat-soluble antioxidants and are one of the breeding goals to improve oil quality. Tocopherols accumulate slowly during 12–41 DAP and reach a maximum concentration during 41-53 DAP in developing seeds of *B. napus*[37]. It has been suggested that production of tocopherols during seed development might be needed for the protection of polyunsaturated fatty acids against peroxidation [38]. In cluster II (21% of genes with transcript abundance differences in time) and cluster III (31% genes) transcript abundance increased gradually or abruptly at 35–40 DAP, respectively (Figure 3). In these clusters, cytochrome P450, late embryogenesis abundant proteins (LEA), LTP (lipid transfer protein) and storage proteins, and abscisic acid and ethylene (hormone metabolism) were over-represented. This observation is in agreement with a number of other studies where storage proteins, abscisic acid and ethylene were highly expressed during late seed developmental stages because of their roles in growth and development of seed tissues, accumulation of seed reserves, maturation, desiccation tolerance, induction of seed dormancy and the utilization of storage reserves to support germination [1, 2, 12, 14, 39].

### Gene co-expression patterns associated with genotypic differences, or genotype- and temporal differences

WGCNA analysis organized 3169 probes associated with genetic variation into 17 gene modules (3169 probes) (Additional file 8: Table S5; Additional file 9: Figure S4A), which could be represented by three gene clusters (cluster IV to VI) through hierarchical clustering (Figure 6). These clusters reveal genetic variation in patterns of transcript abundance during seed development, with distinct variation between the two parents with many genes showing transgressive segregation in DH lines.

Similarly, sets of genes (555 probes) displayed variation in transcript abundance due to both genotype and time contrasts in six gene modules (Additional file 8: Table S5; Additional file 9: Figure S4C). Four different patterns were identified in hierarchical clustering, mainly either with a gradual decrease in transcript abundance from early stages to late stages or a continuous increase across seed development (Figure 7). The leafy-type PC175 usually showed different patterns of transcript abundance compared to the other three genotypes (Figure 7A, 7C-E, 7G-H), while variation in transcript abundance of the two DH lines is more similar to that of the maternal genotype YS143. This could be due to maternal effects on seed and seed characteristics, as reported before in another study [11].

### Predicting *cis*-regulatory elements for co-expressed genes related to lipid metabolism

*Brassica* species are widely cultivated for seed oil, and seed oil is also a major source of energy during germination and seedling growth. Thus, we want to get an insight in the genetic regulation of lipid metabolism in both oil- and vegetable- morphotypes. First, we defined pathways, such as FA synthesis and elongation, FA desaturation, lipid degradation, triacylglycerol. The co-expression analysis identified clusters of genes in the respective pathways with different transcript abundance. For example, FA synthesis and elongation related genes shared a similar time-dependent (high at 18–25 DAP, decrease thereafter) and a genotype-dependent transcript abundance (Additional file 12: Figure S7A). Lipid degradation related genes showed four different patterns of transcript abundance. However, triacylglycerol and FA desaturation biosynthesis processes were highly conserved with similar transcript abundance, increasing during late stages or early to middle stages of development respectively, among all four studied genotypes (Additional file 12: Figure S7B, D).

All these different sets of co-expressed genes in different pathways can be regulated by common or specific regulatory elements. The prediction of putative regulatory elements in co-regulated genes can increase our understanding of seed development and results in tools to breed for improved oil content. Transcription factors play regulatory roles not only in seed development but also in lipid metabolism [40] and transcription factor binding sites (or *cis*-regulating elements) are usually located in upstream regulatory regions of genes.

The ABI4 binding motif was shared by genes from the triacylglycerol biosynthesis pathway, FA desaturation and lipid degradation (Additional file 12: Figure S7C: III-IV), which were all up-regulated 25 DAP. Motif ABI4 was reported as an important *cis*-regulator of the DGAT gene of triacylglycerol biosynthesis [39, 41] and repressor of lipid degradation [42], and is known for its role during seed maturation, seed size, seed germination and seedling growth. The AAAG binding domain was conserved in motifs Dof2, Dof3, PBF and MNB1A (DOF family) and was found specifically in triacylglycerol biosynthesis genes in our seed samples. The roles of DOF genes are in activating seed storage protein genes during seed development and germination in rice [43], barley [44], maize [45], wheat [44] and *Arabidopsis*[46]. The interwoven connection of different regulatory motifs in Figure 5 supports the fact that target genes are regulated by multiple interacting TFs. The interaction between Dof proteins and HMG proteins was reviewed in maize seed [47]. Similarly, the other identified motifs, in this study, that belong to the bZIP, MADS-box, MYB family, beta-beta-alpha zinc finger families, as well as unknown motifs, likely play roles in regulating gene expression during seed development and maturation in *B. rapa*. Some motifs reported in *Arabidopsis* seed that are similar to our findings, such as AG, ABI4, squamosa, bZIP and PEND for triacylglycerol biosynthesis genes, and HMG-1 and Gamyb for FA synthesis genes [7]. Moreover, they also reported many more motifs than our findings, and in addition, several motifs observed for triacylglycerol biosynthesis in our study were reported for FA synthesis in this study or vice versa. The possible explanations for finding different numbers of motifs with some disagreement could be (i) the sequence form 1000 bp upstream plus the UTR region was used by [7], but we considered only 1000 bp upstream sequences because the majority of *cis*-regulatory elements are located in this region [48], and (ii) the use of different motif finding tools; TFBS [49] and fdrMotif [50] by [7] but MEME tool [51] in this study. The different tools use different algorithms and that could lead to some differences in finding motifs [50]. Besides the UTR region and the 1000 bp upstream region, *cis*-regulatory elements can also be located in the downstream sequence, in the gene’s introns or in neighbouring genes’ introns [52] and consideration of these genomic regions can potentially improve in finding TFs binding motifs.

## Conclusions

A morphological characterization of developing embryos and seeds of two different morphotypes of *Brassica rapa,* a pak choi and a yellow sarson, showed that the seed developmental stages based on the shape of the embryo were similar in both morphotypes, but the colour of embryo and seed coat differed at both earlier (15–25 DAP) and later stages (after 40 DAP). Analysis of transcript abundance measured with qRT-PCR of ten selected genes from different metabolic processes suggested to use six time points (18, 20, 25, 30, 35 and 40 DAP) for a global gene expression study using microarrays. In this study, done on pak choi, yellow sarson, and two doubled haploid lines from their cross we found that most changes in transcript abundance occur between 25 and 35 DAP, suggesting that the timing of metabolic processes during seed development in *B. rapa* is later than in *B napus*. We identified 47 gene modules of which 17 showed genotypic variation in transcript abundance, 4 showed temporal variation and 6 showed both temporal and genotypic variation. This study shows that temporal transcriptional variation is more dominant than morphotype or genotype differences. Since lipids are the major storage compounds of *Brassica* seeds, we investigated putative *cis*-regulatory elements of co-regulated gene clusters involved in lipid metabolism. In total 17 putative *cis*-regulatory elements were predicted in 1000 bp upstream region, which are either specific for or common to four co-regulated gene clusters. This study provides detailed information on transcriptional changes during *Brassica* seed development and provides a starting point for a genetical genomics study of seed quality traits.

## Methods

### Plant materials and monitoring seed development

For this study two different *B. rapa* morphotypes were used; an oil-type yellow sarson (YS143) and a vegetable-type pak choi (PC175), as well as two DH lines (DH42 and DH78) from a cross of parental genotypes YS143 and PC175. These two parental morphotypes were selected based on their genetic distance, different plant phenology, flowering time and metabolite content in the seed (Additional file 14: Table S6). The two progeny DH lines, which also differ in morphological characteristics such as seed colour, flowering time and metabolite content were also included in this study (Additional file 14: Table S6). Three plants of parental genotypes and a single plant of each DH line was grown in a heated greenhouse under 16/8 hours light/dark from February to June, 2010 at Wageningen UR. Flowers were tagged the day they opened, assuming self-pollination on the day of flower opening. PC175 and other self-incompatible DH lines of the population were manually bud pollinated to get enough seed. For each genotype, siliques were harvested at 15 time points: 10, 15, 16, 17, 18, 20, 21, 25, 30, 35, 40, 45, 50, 55 and 60 DAP. About 100–150 seeds were excised from the seed pods, frozen in liquid nitrogen and used for RNA isolation. Randomly five seeds from each genotype at each time point (developmental stage) were dissected under the binocular stereo microscope at 1.6x magnification and pictures were taken using Axio Vision Rel. 4.8 software (Carl Zeiss Imaging Solutions, Wrek, Göttingen, Germany) to observe the morphological characteristics of embryos and seeds at each time point.

### RNA isolation

Siliques harvested at defined stages were kept in liquid nitrogen (−196°C), and around 100–150 seeds were extracted under dry ice and ground in liquid nitrogen (−196°C). For real-time PCR, RNA was isolated using KingFisher Flex system (Thermo Scientific, Finland) and Ambion’s MagMAX™-96 Total RNA isolation kit according to the manufacturer’s instruction and RNA pellets were dissolved in nuclease-free water. For microarray, RNA isolation was done using Trizol reagent according to the manufacturer’s instructions (Invitrogen, Burlington, ON, Canada) followed by DNase treatment (AmpGrade I, Invitrogen, Burlington, ON, Canada) and a purification step (RNeasy Mini Kit, Qiagen). The quantity of RNA was determined by NanoDrop ND-100 UV–VIS spectrophotometer and quality was assessed by A260/A280 and A260/A230 ratio (NanoDrop Technologies, Inc., Wilmington, DE, USA) as well as by 1% agarose gel.

### Quantitative real-time PCR (qRT-PCR)

Ten genes involved in major metabolic processes of seed development according to the literature were selected to measure transcript abundance across seed development stages ranging from 10 to 60 DAP using real time-PCR (Additional file 15: Table S7). These candidate genes represent fatty acid biosynthesis (DGAT1, DGAT2 and FAE1), carbohydrate metabolism (GBSSI and SuSy3), storage proteins (12S-CRA1 and LEA), transcription factors (LEC1 and Glabra2) and one CHD3-chromatine-remodeling factor (PICKLE). The detailed procedure of qRT-PCR and normalization is described in Additional file 16. The normalized transcript abundance (∆∆CT) of each gene for each sample was determined with respect to the reference gene β-actin. We use the term gene expression for this normalized transcript abundance in this paper. In order to identify common profiles of transcript abundance across the seed development stages, genes were grouped using hierarchical cluster analysis with Euclidean distance of normalized data (∆∆CT). Transcript abundance of ten genes obtained from real-time PCR were visualized using a heatmap tool in Additional file 1: Figure S1.

### Microarray probe design

The whole genome sequence of *B. rapa* cv. Chiifu (a leafy vegetable inbred line) is publicly available [27]. We designed microarray probes for two-colour Agilent microarray platform based on the predicted gene models of the reference genome sequence. In this custom array, 61,654 probes were assembled, which represent 40,879 (99.74%) *B. rapa* gene IDs (Bra ID) and 108 (0.26%) scaffold IDs with no assignment of Bra ID (Additional file 2: Table S1). All the probes were annotated into 35 different functional categories or “BINS” as defined by MapMan software (Additional file 17). MapMan is an open source software tool to categorize and display functional genomics data [53].

### Experimental design for microarray hybridization

Microarray hybridization was done on developing seeds from four genotypes; the two parents (YS143 and PC175) and two DH lines (DH42 and DH78) at six time points: 18, 20, 25, 30, 35 and 40 DAP. Two independent experiments were done to compare two parental genotypes (hereafter, called experiment A) and two DH lines (hereafter, called experiment B). Cy3 and Cy5 dyes were incorporated into cRNA samples according to the Agilent two-colour microarray based gene expression analysis (Low input quick Amp labelling G4140-90050) protocol (Agilent Technologies, Inc., Santa Clara, CA, USA) and hybridized on arrays following a double-loop design (Additional file 18: Figure S9A-B). In one array, two samples from the two consecutive time points of the same genotype or two genotypes from the same time point were hybridized. The same hybridization scheme was used for experiment B using the two DH lines. In both experiments A and B, each sample was hybridized four times generating four technical replicates. Loess was used for within-array normalization and quantile normalization for between-array normalization using the limma package in R [54]. The normalized Cy3 and Cy5 intensities were used as measures of transcript abundance and are sometimes referred to as gene expression in this paper.

### Microarray data analysis

The aim of this study was to explore the effects of seed developmental stages, genotypic variation or both on transcript abundance of genes with special focus on important metabolic processes. Principal components analysis (PCA) was used to examine the global profiles of transcript abundance of the four *B. rapa* genotypes across six seed developmental stages.

For further analyses, we excluded probes with little variation in transcript abundance across seed development as well as between genotypes using a minimum two-fold change threshold (in absolute value). Fold change differences were calculated in contrasts between two consecutive time points (18 vs. 20, 20 vs. 25, 25 vs. 30 and 35 vs. 40 DAP) as well as between two pairs of genotypes (YS143 vs. PC175 and DH42 vs. DH78) per time point. In this study, we emphasized the metabolic processes that have either a high number of selected probes or apparent changes in the number of selected probes among time point or genotype contrasts for further analysis.

WGCNA is a widely used correlation-based network construction method to construct a scale-free network [36]. A signed WGNCA approach was applied in this study to find gene co-expression modules, so-called “gene modules” while keeping track of positive or negative correlation coefficients, where each gene module represents a group of genes having similar co-expression patterns across seed developmental stages or genotypes or their combinations. WGCNA first calculates Pearson’s correlation matrix of all genes, and transforms the correlation matrix into an adjacency matrix by raising all values to a soft threshold power β (default value 12) to emphasize strong correlations and penalize weaker correlations on an exponential scale. Then, the adjacency matrix is transformed into a topological overlap matrix (TOM), which summarizes the degree of shared connections between any two genes, and then converted into a dissimilarity matrix. A hierarchical cluster of genes is created based on a dissimilarity matrix and finally, gene co-expression modules were defined from the cluster dendrogram at a threshold of 0.2 dissimilarity value using the dynamic tree-cutting algorithm. Once gene modules were identified, the “Module Eigengene” (ME; the first principal component of the expression values across subjects) was calculated using all probes in each gene module. The module eigengene represents the expression profiles of all probes from a gene module across subjects (i.e. genotypes at each time point), and high or low eigengene values of subjects correspond to over- or under expression in the corresponding subjects, respectively. The details of this method are described in [36, 55], and the analysis was performed in R software using the WGCNA package [56]. The module eigengene of each subject was examined to determine the effects of time or genotype or both using an ANOVA test. In this case, genotype and time were two independent factors and a module’s eigengene values as the response, consecutively for each module. The significance of the effects was determined at 0.001 FDR correction proposed by [57]. The probes belonging to gene modules significant in ANOVA were grouped into three categories according to genotype or time or both genotype and time effect. Hierarchical clustering using Euclidean distance as a criterion for dissimilarity then was applied independently on the data sets of these three categories. From this hierarchical clustering, genes were broadly organized into clusters considering the height of the dendrogram, and each category was annotated with MapMan metabolic pathways. Fisher’s exact test was used to test for over- and under-representation of metabolic pathways in a selected cluster of genes using R software. If a particular pathway was significantly over- or under-represented in the gene cluster that indicates a statistically significant number of probes from the pathway are present in the gene clusters with specific patterns of gene expression across seed development stages over four genotypes [58].

### Motif analysis

We focused on discovering transcription factor binding sites or DNA motifs for the co-expressed genes of lipid metabolism. The 1000 bp upstream sequences of co-expressed *Brassica* genes from the transcription start site (TSS) were retrieved from *Brassica* database (http://brassicadb.org/brad/). Conserved DNA motifs were searched in the upstream regions using the expectation maximization algorithm implemented in MEME version 4.9.0 [51]. Motifs with 6–12 nucleotides length were searched on both strands of the input sequence using both “zero or one occurrence per sequence” and “any number of repetitions” options. Motifs with and E-value ≤ 1 were used to assess similarity to known motifs using TOMTOM [59] in the JASPAR plant specific database [60]. This plant specific JASPAR database was considered because of the potential roles of these motifs in regulating lipid metabolism during seed development in higher plants.

### Availability of supporting data

The data sets supporting the results of this article are included within the article and its additional files.

## Electronic supplementary material

Additional file 1: Figure S1: Transcript abundance profiles of ten genes used in real-time PCR gene expression. FAE1, DGAT1, DGAT2 from lipid metabolism, SUS3 and GBSSI from carbohydrate metabolism, 12S-CRA1 and LEA from storage proteins, LEC1 and Glabra2 are transcription factors and PICKLE as CHD3-chromatine-remodeling factor. The red colour indicates a high abundance level, green a low level, and grey for missing values. Vertical white lines separate genotypes, yellow-coloured square boxes mark three different groups with high abundance level. Purple coloured boxes at the top indicate that those time-points were selected for the later microarray experiments. (TIFF 251 KB)

Additional file 2: Table S1: Number and percentage of Brassica ID (Bra ID) represented in the microarrays, annotated according to MapMan defined metabolic processes. (ODS 19 KB)

Additional file 3: Figure S2: Pearson correlation coefficients between time points with four replicates per time point within each genotype using all 61654 microarray probes. **A**. Upper triangle: PC175, lower triangle: YS143. **B**. upper triangle: DH78, lower triangle: DH42. (TIFF 473 KB)

Additional file 4: Table S2: Spearman correlation coefficients between real-time PCR and microarray transcript abundance profiles across genotype and seed developmental stages. (ODS 13 KB)

Additional file 5: Figure S3: Number of probes associated with early stages 18–25 DAP (< −0.01 PC1 loadings) and late stages 35–40 DAP (> 0.01 PC1 loadings) in principal components analysis (PCA). The probes were classified according to MapMan functional categories. (TIFF 253 KB)

Additional file 6: Table S3: List of selected probes with genotype contrasts in two experiments A and B, as well as time point contrasts in all four genotypes using a minimum 2-fold change criterion. (ODS 217 KB)

Additional file 7: Table S4: Number of selected probes ( > absolute 2 fold-change criteria) from temporal contrasts and genotype contrasts into MapMan functional categories. Metabolic processes in highlighted cells are used for further analysis because of apparent changes in the number of selected probes. (ODS 20 KB)

Additional file 8: Table S5: WGCNA gene modules with a significant association with genotypes or time or both genotype and time in ANOVA analyses. The threshold for the level of significance was set at the 0.001 FDR level (Benjamini and Hochberg method). The highlighted cells indicate significant gene modules selected for further analysis. (ODS 20 KB)

Additional file 9: Figure S4: Representative abundance levels of gene transcripts belonging to 27 WGCNA gene modules that are significantly associated with **A**. Genotypic differences **B**. temporal differences (time points) **C**. both genotypic differences and temporal differences. Horizontal solid lines separate gene modules and vertical dashed lines separate genotypes. Time points are in ascending order in all genotypes. Numbers in the left corner represent gene modules. (TIFF 535 KB)

Additional file 10: Figure S5: Over- and under- representation analysis of time dependent clusters (Cluster I, II and III) into MapMan functional categories using Fisher’s exact test. Pink to red colour indicates increasing significance levels for overrepresentation and purple to blue colour increasing significance levels for under-representation. The darker the colour intensity, the more significant. Only significance levels with p < 0.05 after FDR correction with the Benjamini-Hochberg method are highlighted. The horizontal green lines separate different pathways. (TIFF 597 KB)

Additional file 11: Figure S6: General overview of lipid metabolism showing fatty acid (FA) biosynthesis, FA elongation, lipid desaturation, TAG biosynthesis and glycolipid biosynthesis. (TIFF 143 KB)

Additional file 12: Figure S7: Heatmap of gene expression values with hierarchical clustering (Euclidean distance) of all the selected probes (> (> absolute 2-fold change) belonging to **A**. Fatty acid (FA) synthesis and elongation **B**. FA desaturation **C**. FA degradation **D**. triacylglycerol (TAG) biosynthesis pathways of lipid metabolism. Vertical white bars separate genotypes (YS: yellow sarson, PC: pak choi, DH42: DH line 42 and DH78: DH line 78). Time points are arranged in ascending order from 18 to 40 DAP within each genotype. (TIFF 613 KB)

Additional file 13: Figure S8: Over- and under- representation analysis of gene clusters IV, V and VI, that showed genotypic differences in expression patterns, into MapMan functional categories using Fisher’s exact test. Pink to red colour indicates increasing significance levels of overrepresentation and purple to blue colour for increasing significance levels for underrepresentation. The darker the colour intensity, the more significant. Only significance levels with p < 0.05 after FDR correction with the Benjamini-Hochberg method are highlighted. The horizontal green lines separate different pathways. (TIFF 654 KB)

Additional file 14: Table S6: Morphological and metabolic descriptions of two parental and two doubled haploid genotypes. (ODS 12 KB)

Additional file 15: Table S7: List of genes used for real-time PCR with their gene name, primer sequence (forward and reverse primers), melting temperature (Tm), GC content percentage, metabolic process and gene ontology biological process (BP). (ODS 16 KB)

Additional file 16: **Methods used for quantitative real-time PCR.** (DOCX 17 KB)

Additional file 17: **Method used for annotation of microarray probes into MapMan functional categories.** (DOCX 16 KB)

Additional file 18: Figure S9: Double loop design for hybridization of samples on two-colour Agilent microarrays. Sample names are a combination of genotypes (YS = yellow sarson, PC = pak choi, 42 = DH line 42 and 78 = DH line 78) and time points (18, 20, 25, 30, 35 and 40 Days after pollination). The colours of the arrows in the loop indicate Cy3 (green) and Cy5 (red) dyes in this microarray experiment. **A**. Experiment A represents the design for hybridization of the parental genotypes (yellow sarson and pak choi). **B**. experiment B for the two DH lines (DH42 and DH78). (TIFF 338 KB)
