# Supplementary figures and images for: Genome-wide analysis of coordinated transcript abundance during seed development in different Brassica rapa morphotypes
Source: BMC Genomics. 2013 Dec 1;14(1):840. doi: 10.1186/1471-2164-14-840 (PMC4046715; doi:10.1186/1471-2164-14-840)

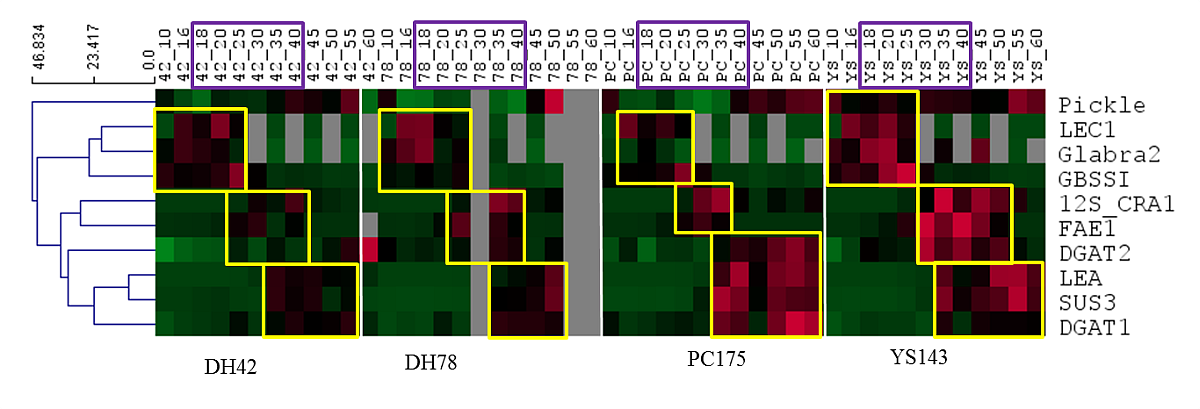

Supplement: Supplementary file 1 — Additional file 1: Figure S1: Transcript abundance profiles of ten genes used in real-time PCR gene expression. FAE1, DGAT1, DGAT2 from lipid metabolism, SUS3 and GBSSI from carbohydrate metabolism, 12S-CRA1 and LEA from storage proteins, LEC1 and Glabra2 are transcription factors and PICKLE as CHD3-chromatine-remodeling factor. The red colour indicates a high abundance level, green a low level, and grey for missing values. Vertical white lines separate genotypes, yellow-coloured square boxes mark three different groups with high abundance level. Purple coloured boxes at the top indicate that those time-points were selected for the later microarray experiments. (TIFF 251 KB) [file 12864_2013_5564_MOESM1_ESM.tiff]

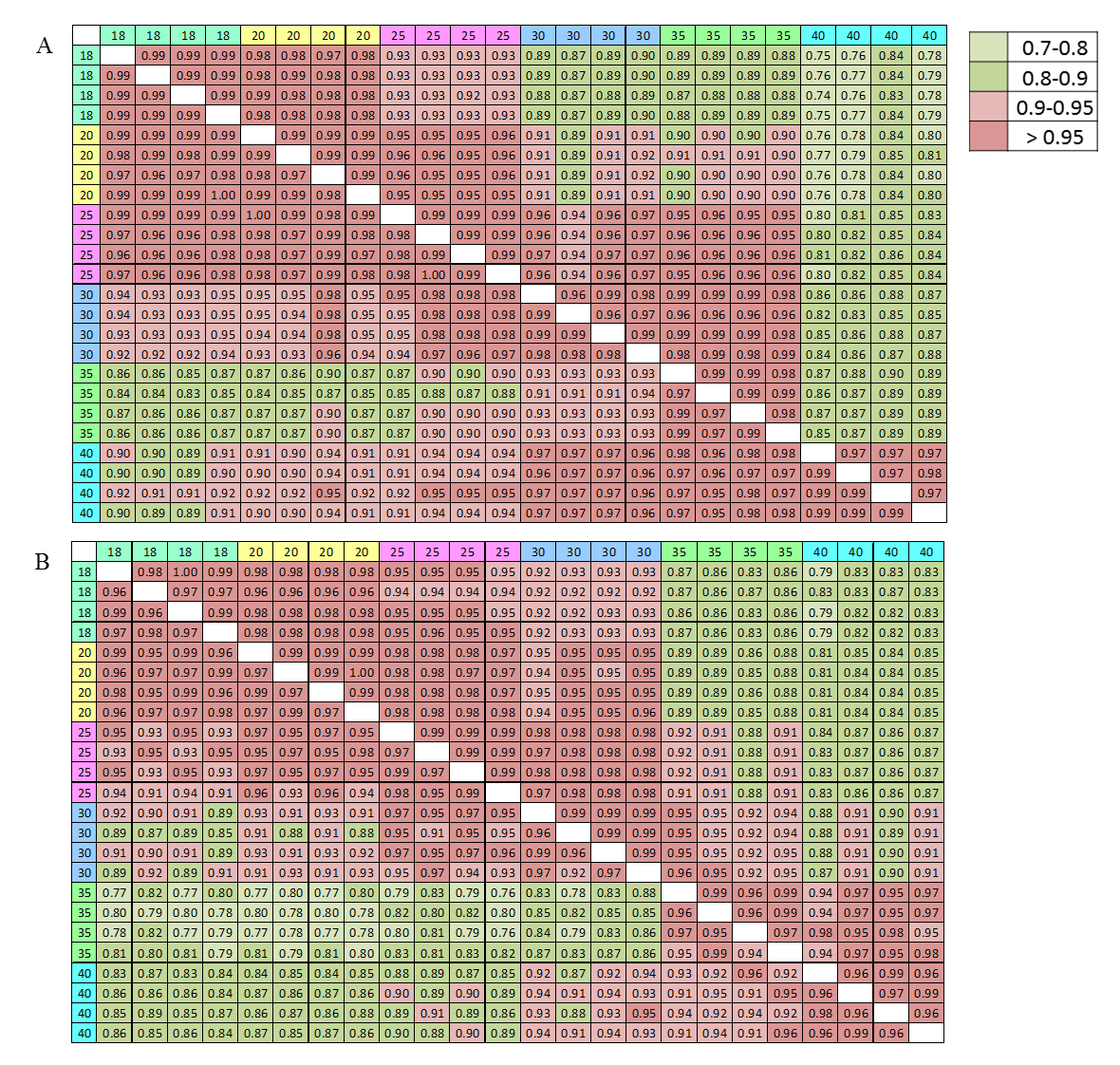

Supplement: Supplementary file 3 — Additional file 3: Figure S2: Pearson correlation coefficients between time points with four replicates per time point within each genotype using all 61654 microarray probes. A. Upper triangle: PC175, lower triangle: YS143. B. upper triangle: DH78, lower triangle: DH42. (TIFF 473 KB) [file 12864_2013_5564_MOESM3_ESM.tiff]

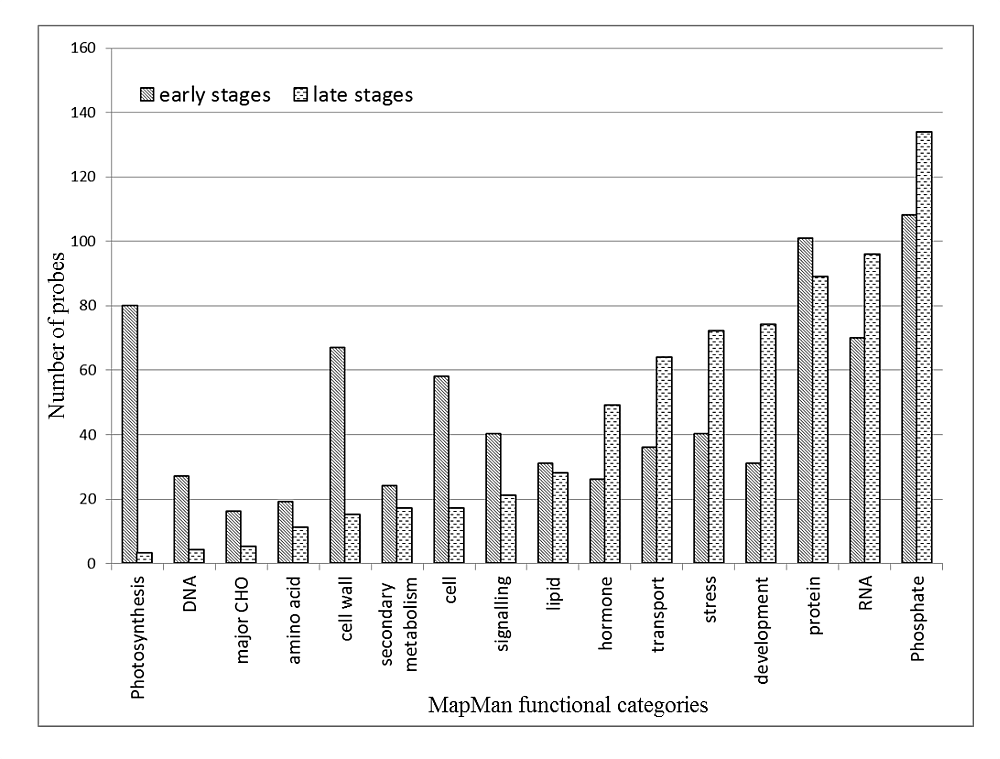

Supplement: Supplementary file 5 — Additional file 5: Figure S3: Number of probes associated with early stages 18–25 DAP (< −0.01 PC1 loadings) and late stages 35–40 DAP (> 0.01 PC1 loadings) in principal components analysis (PCA). The probes were classified according to MapMan functional categories. (TIFF 253 KB) [file 12864_2013_5564_MOESM5_ESM.tiff]

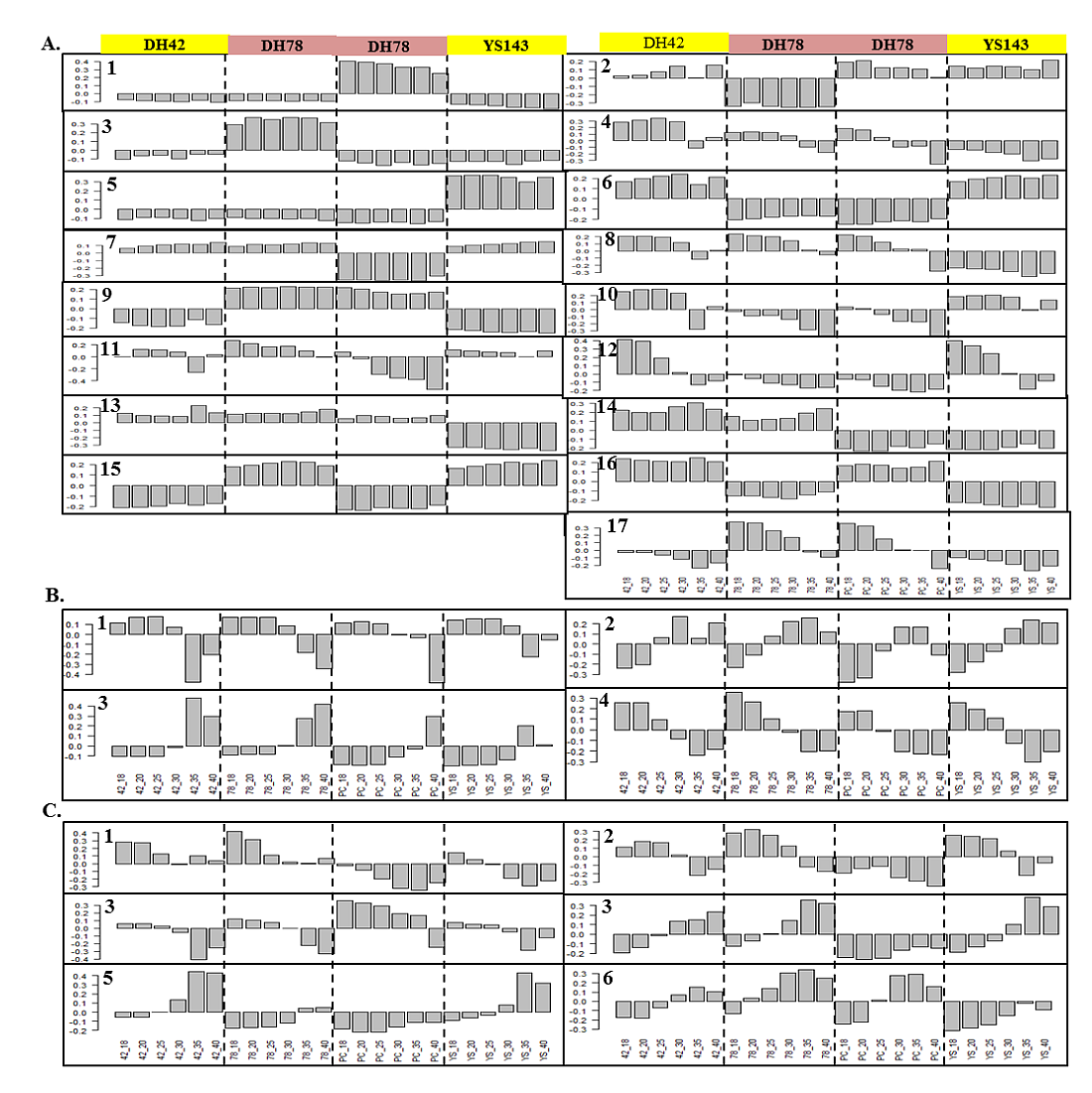

Supplement: Supplementary file 9 — Additional file 9: Figure S4: Representative abundance levels of gene transcripts belonging to 27 WGCNA gene modules that are significantly associated with A. Genotypic differences B. temporal differences (time points) C. both genotypic differences and temporal differences. Horizontal solid lines separate gene modules and vertical dashed lines separate genotypes. Time points are in ascending order in all genotypes. Numbers in the left corner represent gene modules. (TIFF 535 KB) [file 12864_2013_5564_MOESM9_ESM.tiff]

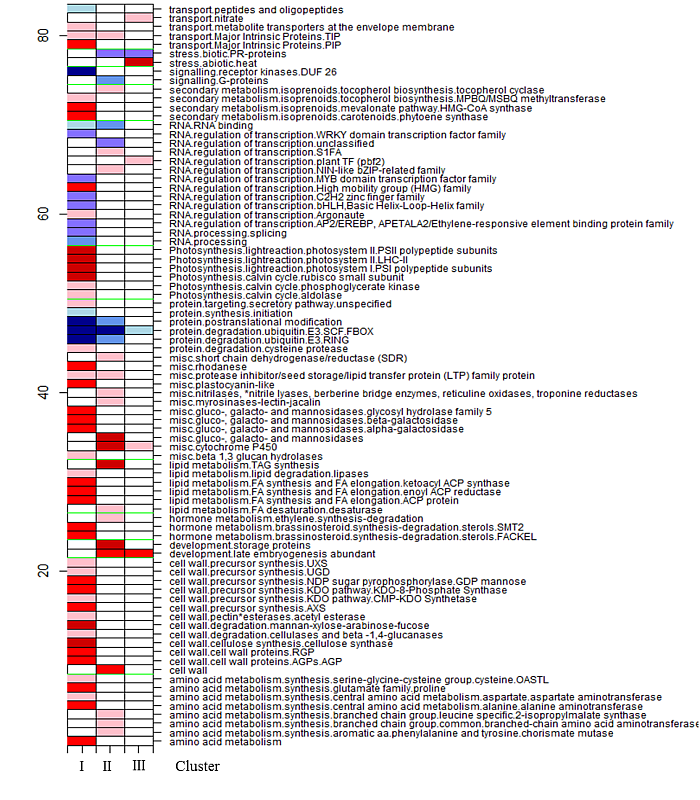

Supplement: Supplementary file 10 — Additional file 10: Figure S5: Over- and under- representation analysis of time dependent clusters (Cluster I, II and III) into MapMan functional categories using Fisher’s exact test. Pink to red colour indicates increasing significance levels for overrepresentation and purple to blue colour increasing significance levels for under-representation. The darker the colour intensity, the more significant. Only significance levels with p < 0.05 after FDR correction with the Benjamini-Hochberg method are highlighted. The horizontal green lines separate different pathways. (TIFF 597 KB) [file 12864_2013_5564_MOESM10_ESM.tiff]

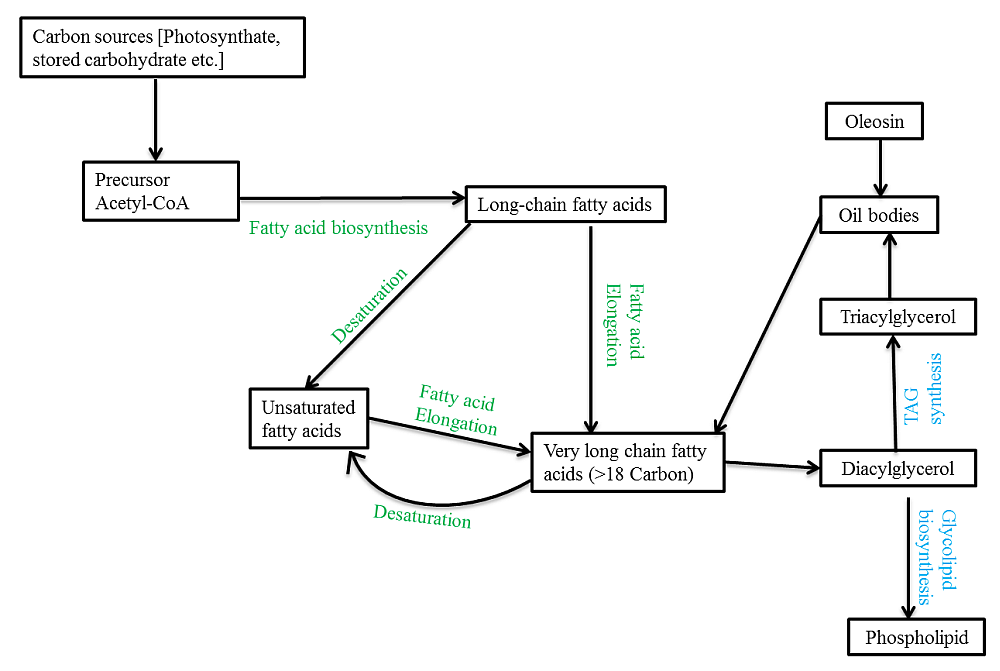

Supplement: Supplementary file 11 — Additional file 11: Figure S6: General overview of lipid metabolism showing fatty acid (FA) biosynthesis, FA elongation, lipid desaturation, TAG biosynthesis and glycolipid biosynthesis. (TIFF 143 KB) [file 12864_2013_5564_MOESM11_ESM.tiff]

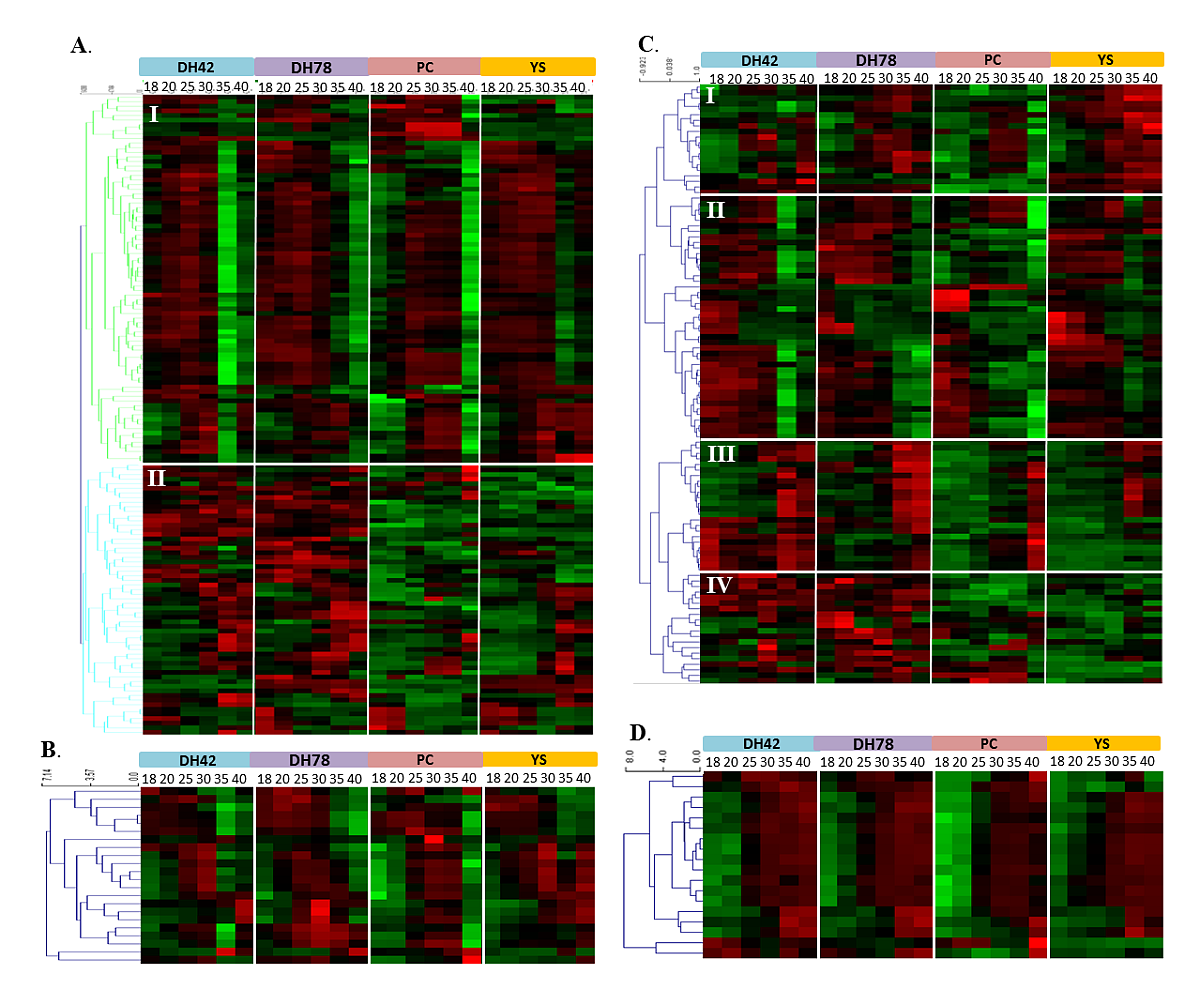

Supplement: Supplementary file 12 — Additional file 12: Figure S7: Heatmap of gene expression values with hierarchical clustering (Euclidean distance) of all the selected probes (> (> absolute 2-fold change) belonging to A. Fatty acid (FA) synthesis and elongation B. FA desaturation C. FA degradation D. triacylglycerol (TAG) biosynthesis pathways of lipid metabolism. Vertical white bars separate genotypes (YS: yellow sarson, PC: pak choi, DH42: DH line 42 and DH78: DH line 78). Time points are arranged in ascending order from 18 to 40 DAP within each genotype. (TIFF 613 KB) [file 12864_2013_5564_MOESM12_ESM.tiff]

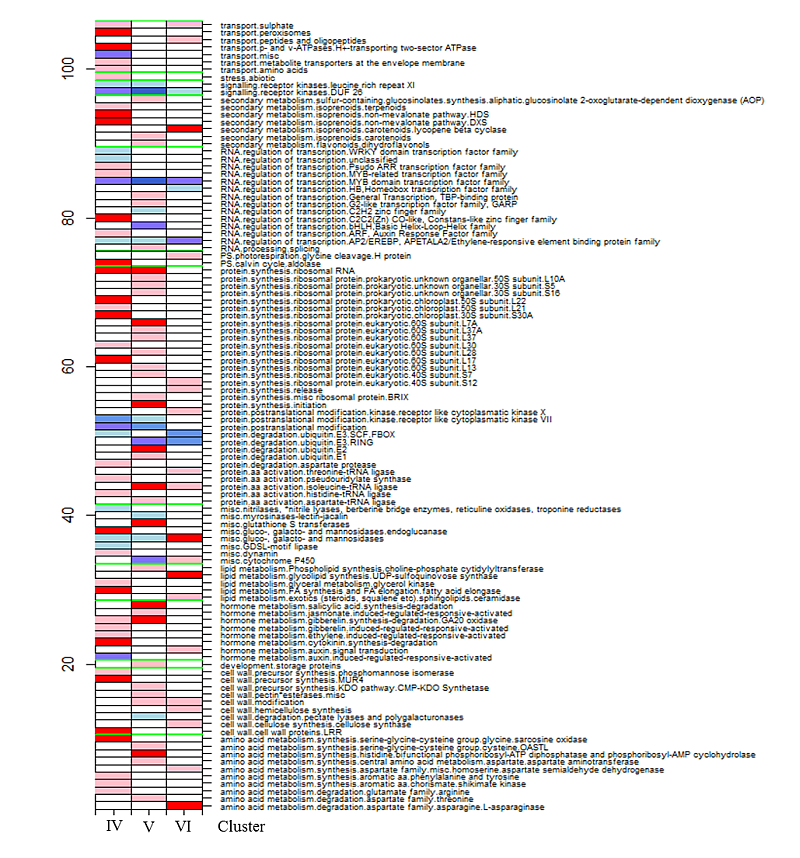

Supplement: Supplementary file 13 — Additional file 13: Figure S8: Over- and under- representation analysis of gene clusters IV, V and VI, that showed genotypic differences in expression patterns, into MapMan functional categories using Fisher’s exact test. Pink to red colour indicates increasing significance levels of overrepresentation and purple to blue colour for increasing significance levels for underrepresentation. The darker the colour intensity, the more significant. Only significance levels with p < 0.05 after FDR correction with the Benjamini-Hochberg method are highlighted. The horizontal green lines separate different pathways. (TIFF 654 KB) [file 12864_2013_5564_MOESM13_ESM.tiff]

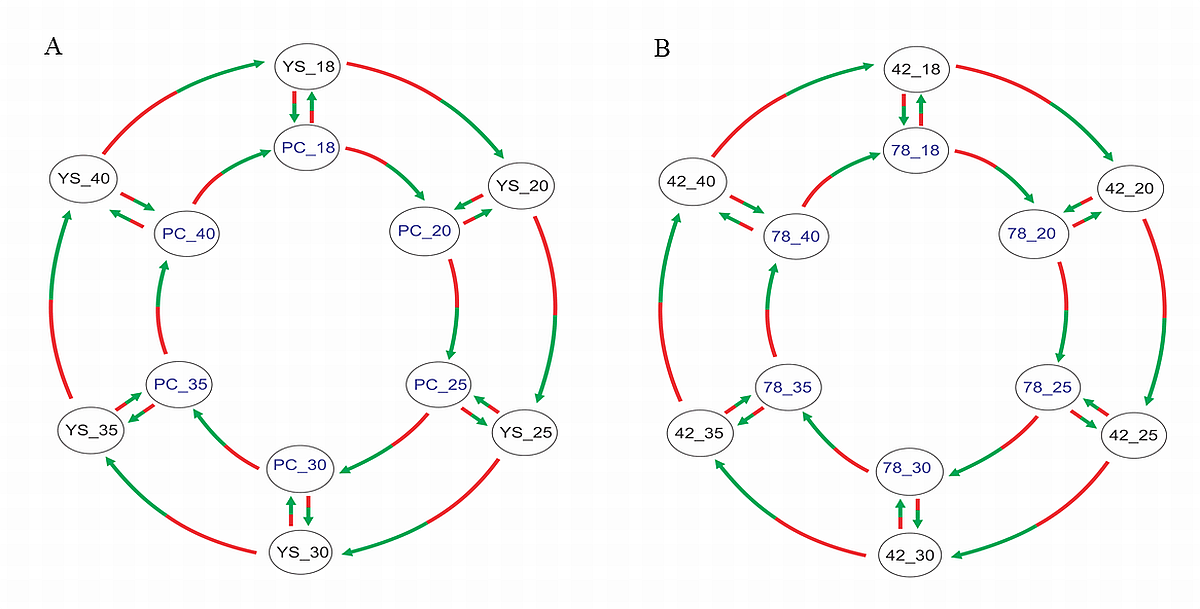

Supplement: Supplementary file 18 — Additional file 18: Figure S9: Double loop design for hybridization of samples on two-colour Agilent microarrays. Sample names are a combination of genotypes (YS = yellow sarson, PC = pak choi, 42 = DH line 42 and 78 = DH line 78) and time points (18, 20, 25, 30, 35 and 40 Days after pollination). The colours of the arrows in the loop indicate Cy3 (green) and Cy5 (red) dyes in this microarray experiment. A. Experiment A represents the design for hybridization of the parental genotypes (yellow sarson and pak choi). B. experiment B for the two DH lines (DH42 and DH78). (TIFF 338 KB) [file 12864_2013_5564_MOESM18_ESM.tiff]
