# Supplementary material for: Genome-wide analysis of coordinated transcript abundance during seed development in different Brassica rapa morphotypes
Source: BMC Genomics. 2013 Dec 1;14(1):840. doi: 10.1186/1471-2164-14-840 (PMC4046715; doi:10.1186/1471-2164-14-840)
Supplement: Supplementary file 16 — Additional file 16: Methods used for quantitative real-time PCR. (DOCX 17 KB) [file 12864_2013_5564_MOESM16_ESM.docx]

**Additional file 2:**

**Materials and methods**

**Quantitative real-time PCR, primer design and normalization procedure**

All the RNA samples were adjusted to the same concentration (50 ng/µl) for a final volume of 9 µl and DNAse treatment was done with DNAse I kit (Invitrogen; Burlington, Canada). cDNA synthesis was performed with iScriptTM kit (Bio-Rad; Hercules, California, USA) according to the supplier’s instructions and a final volume of 20 µl at around 1000 ng/µl per sample was obtained. cDNA working dilutions were made at 1:20. The cDNA products were stored at -70^0^C. Primers were designed with Beacon Designer v. 7.8 software (Bio-Rad; Premier Biosoft, Palo Alto, California, USA) to amplify PCR products no longer than 200bp without primer dimmer. Primers that give rise in PCR reactions using cDNA template to primer dimers, and have poor melting curves and primer efficiency, were discarded. The primer efficiency was calculated from the relative fluorescence units at each temperature for each well with LingReg v.1.1.23 software [1]. Primer efficiency was, in general, close to 2, and hence, we assumed primer efficiency 2 in subsequent analyses. The qRT-PCR reactions were performed in a CFX 96^TM^ real-time PCR Detection System (Bio-Rad; Hercules, California, USA) using iQ^TM^ SYBR Green Supermix reagent (Bio-Rad; Hercules, California, USA). A standard reaction volume (20 µl per reaction) contained 4 µl cDNA template, 5 µl SYBR Green Supermix, 2.4 µl RNA free MQ water, 0.3 µl forward and reverse primers (10 µM). The PCR protocol consisted of and initial denaturalization step at 95^0^C for 3 minutes, followed by 40 cycles of 95^0^C for 15 seconds and 60^0^C for 1 minute.

Among β-actin, α-tubulin and ubiquitin C, β-actin gene was chosen as a reference gene due to its stability in expression across time points and genotypes. Two technical replicates of PCR reaction per sample were prepared for each gene, where a sample represents a specific genotype with specific time point (DAP), and run in a 96-well plate. Samples with more than 0.25 standard deviation (CT) values between technical replicates were discarded. The relative expression (∆CT) and normalized expression (∆∆CT) of each gene for each sample was determined with respect to reference gene β-actin using the method described by [2], implemented in the CFX Manager Software (Bio-Rad, USA). Hierarchical cluster analysis with Euclidean distance and heatmap tool were used to analyze the gene transcript abundance across the seed development stages.

**References**

1. Ramakers C, Ruijter JM, Deprez RHL, Moorman AFM: **Assumption-free analysis of quantitative real-time polymerase chain reaction (PCR) data**. *Neuroscience Letters* 2003, **339**(1):62-66.

2. Pfaffl MW: **A new mathematical model for relative quantification in real-time RT–PCR**. *Nucleic Acids Research* 2001, **29**(9):e45.
