# Supplementary material for: Genome-wide analysis of coordinated transcript abundance during seed development in different Brassica rapa morphotypes
Source: BMC Genomics. 2013 Dec 1;14(1):840. doi: 10.1186/1471-2164-14-840 (PMC4046715; doi:10.1186/1471-2164-14-840)
Supplement: Supplementary file 17 — Additional file 17: Method used for annotation of microarray probes into MapMan functional categories. (DOCX 16 KB) [file 12864_2013_5564_MOESM17_ESM.docx]

**Additional file 3:**

**Materials and methods**

**Annotation of microarray probes**

In MapMan software probe annotation, probes were searched against 6 different databases: The Arabidopsis Information Resource (TAIR8) [1], SwissProt/Uniprot plant proteins (PPAP) [2], Uniref90 Blast database (UNIREF) [3], Clusters of Orthologous Groups (KOG) [4], Conserved Domain Database (CDD) [5], InterProScan and (IPR) [6]. The program used search algorithms; BLASTP for 3 databases (TAIR8, PPAP and UNIREF), RPSBLAST for CDD and KOG, and interProScan. A probe with a database hit bit score more than 50 were assigned into a functional BIN, and probes with a score less than 50 were categorized as “unassigned group”. Probes from category “unassigned group” were excluded in this study.

**References**

1. Swarbreck D, Wilks C, Lamesch P, Berardini TZ, Garcia-Hernandez M, Foerster H, Li D, Meyer T, Muller R, Ploetz L *et al*: **The Arabidopsis Information Resource (TAIR): gene structure and function annotation**. *Nucleic Acids Research* 2008, **36**(suppl 1):D1009-D1014.

2. Schneider M, Bairoch A, Wu CH, Apweiler R: **Plant Protein Annotation in the UniProt Knowledgebase**. *Plant Physiology* 2005, **138**(1):59-66.

3. Suzek BE, Huang H, McGarvey P, Mazumder R, Wu CH: **UniRef: comprehensive and non-redundant UniProt reference clusters**. *Bioinformatics* 2007, **23**(10):1282-1288.

4. Tatusov R, Fedorova N, Jackson J, Jacobs A, Kiryutin B, Koonin E, Krylov D, Mazumder R, Mekhedov S, Nikolskaya A *et al*: **The COG database: an updated version includes eukaryotes**. *BMC Bioinformatics* 2003, **4**(1):41.

5. Marchler-Bauer A, Anderson JB, Derbyshire MK, DeWeese-Scott C, Gonzales NR, Gwadz M, Hao L, He S, Hurwitz DI, Jackson JD *et al*: **CDD: a conserved domain database for interactive domain family analysis**. *Nucleic Acids Research* 2007, **35**(suppl 1):D237-D240.

6. Zdobnov EM, Apweiler R: **InterProScan – an integration platform for the signature-recognition methods in InterPro**. *Bioinformatics* 2001, **17**(9):847-848.
